# Hot carrier generation in a strongly coupled molecule–plasmonic nanoparticle system

**DOI:** 10.1515/nanoph-2022-0700

**Published:** 2023-03-15

**Authors:** Katarzyna Kluczyk-Korch, Tomasz J. Antosiewicz

**Affiliations:** Faculty of Physics, University of Warsaw, Pasteura 5, PL-02-093 Warsaw, Poland

**Keywords:** hot carrier generation, metallic nanoparticle, molecule plasmon coupling, strong coupling

## Abstract

In strongly coupled light matter systems electronic energy levels become inextricably linked to local electromagnetic field modes. Hybridization of these states opens new relaxation pathways in the system, particularly important for plasmon decay into single electron states, known as hot carriers. We investigate the influence of the coupling strength between a plasmonic resonator and a molecule on hot carrier generation using first principles calculations. An atomistic approach allows the capture of changes in the electronic structure of the system. We show that hot carriers are not only preferably generated at excitation frequencies matching the new polaritonic resonances, but their energy distribution strongly deviates from the one corresponding to the non-interacting system. This indicates existence of new plasmon decay paths due to appearance of hybridized nanoparticle–molecule states. We observe also direct electron transfer between the plasmonic nanoparticle and the molecule. Therefore, we may conclude, that bringing plasmonic nanostructures in strong interaction with molecules gives the ability to manipulate the energy distribution of the generated hot carriers and opens possibility for charge transfer in the system.

## 1 Introduction

Localized surface plasmon excitations, i.e. collective electron oscillations confined in nanostructures, may decay in various radiative and non-radiative processes [1, 2]. The non-radiative processes result in a non-equilibrium distribution of highly energetic, hot electrons and holes (hot carriers) in the material. If extracted efficiently, hot carriers may enhance efficiency of variety of devices and processes, e.g. solar cells [3, 4], photoelectrochemical cells [5, 6], photoelectrochemical water splitting [7] and photo-catalysis [8–11].

The induced hot carrier energy distribution is a function of excitation energy, electronic band structure of the material and nanostructure geometry [12, 13]. At the same time, an optical mode such as a localized surface plasmon resonance (LSPR) and an electronic exciton can be coupled to create new hybridised electronic states [14, 15]. Changes in electronic structure of the coupled system occur in the strong coupling regime and can be observed experimentally as a new shifted resonances in photoabsorption spectra [16, 17]. As a result of efficient energy transfer between the subsystems, new hybridised states are created, providing additional decay pathways for excitons in the form of chemical interface damping and chemical interface scattering [10, 18]. Moreover, a large enhancement of the electromagnetic field in a plasmonic cavity affects the hot carrier relaxation time, as recently shown for thin Au films [19]. Similarly, strong coupling between a metal particle’s LSPR and a Fabry–Pérot nanocavity mode in a TiO_2_ thin film was shown to promote electron transfer from the Au nanoparticle (NPs) to TiO_2_ and to enhance the efficiency of the plasmon-induced water-splitting reaction [20, 21].

The coupling strength between two subsystems grows with an increase of the energy transfer rate between the interacting entities [16, 22]. Generally, it is determined by the electromagnetic field lifetime, confinement, and dipole moment. Among the variety of potential cavities, plasmonic structures have received significant attention due to their ability for deep subwavelength focusing of light, considerably reducing their optical mode volume. Atomic scale features on the NPs surface were further shown to confine light even in the picometer scale [23–26]. Despite relatively large losses characteristic for plasmonic nanostructures, room-temperature strong coupling between plasmonic resonantors and excitons in e.g. quantum dots [27] and WSe_2_ [28] or molecules [29] was observed.

Hot carrier generation and hot carrier injection processes have been the subject of numerous works, however, usually only isolated metallic nanostructures made of noble (Au, Ag, Cu) [12, 30], [31], [32], [33], [34] or simple metals (Al, K, Na) [35, 36] were considered. Furthermore, some effort has been directed towards studying hot carriers dynamics between plasmonic nanostructures and adsorbents [37–39] and in core–shell NPs at the metal/metal-oxide interface [5].

Hot carriers are mainly generated in one of four distinct mechanisms: direct interband transitions, impurity or phonon assisted transitions, surface assisted transitions, and momentum conserved formation of multiple electron–hole pairs from one photon [8, 18, 40]. Direct interband transitions are allowed only when the LSPR energy is equal to or greater than the energy gap between involved bands. Nevertheless, in hybrid plasmonic structures, such as core–shell configurations or in the presence of adsorbents, additional energy states may be created allowing for direct transitions to occur [18]. Contribution of geometry assisted transitions generally scales inversely with the size of the NP [18, 41] and is more important for NPs with a large surface to volume ratio. Another factor influencing the hot carrier generation rate is the intensity of the electromagnetic field. Therefore, complex geometries of NPs supporting creation of a large number of intense electromagnetic field spots (so called hot spots) are thought to be promising architectures for efficient hot carrier generators [33, 34].

In this paper, we study the influence of the coupling strength between an optical mode of a metal NP, i.e. the LSPR, and a molecule on the hot carrier energy distribution using first principles time-dependent density functional theory (TDDFT) calculations. The atomistic approach allows for the capture of changes in the electronic structure of the system, in contrast to jellium free-electron-like models, with these changes affecting the hot carrier generation mechanism [32]. The geometry of the investigated structure is treated accurately, so surface assisted effects such as Landau damping are included. We follow individual transitions and visualise the wave functions of the coupled system showing increasing hybridisation resulting from the coupling of the plasmon and the molecular exciton, which manifests itself in the degree of charge transfer between the NP and the molecule.

## 2 Results and discussion

### 2.1 System description and methodology

**Figure 1:**
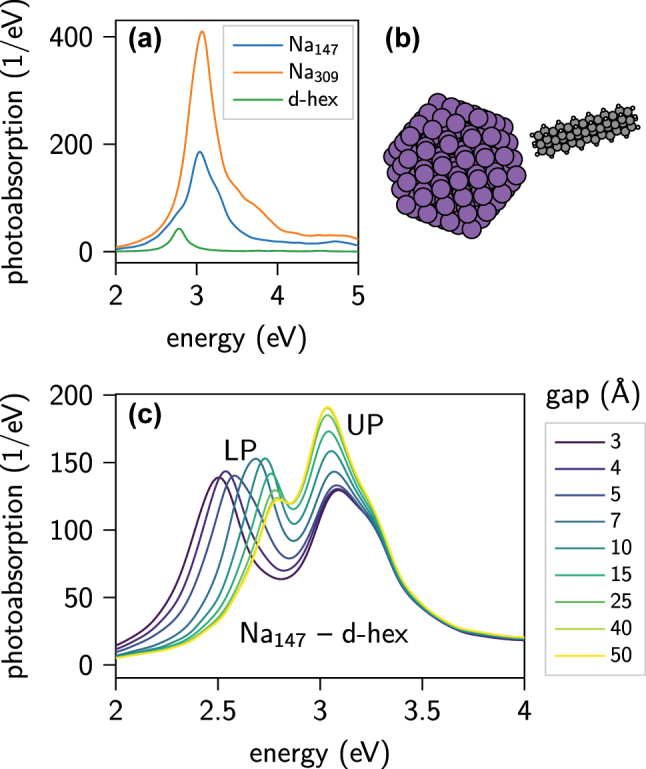
Coupled plasmon-molecule system. (a) Photoabsorption spectra of individual moieties and (b) sketch of the Na_147_-dimer-of-hexacene system. (c) Photoabsorption spectra of the coupled system versus nanoparticle–molecule gap size show a clear evolution of the lower and upper polaritons with gap size. At the largest gap size of 50 Å the two-peaked spectrum corresponds to the uncoupled (incoherent) sum of the Na_147_ and molecular spectra.

We consider a model system of icosahedral Na_147_ and Na_309_ NPs coupled to a dimer of hexacene (d-hex) molecules (see Figure 1(b)). The free-electron-like structure of Na eases the computational efforts and analysis in comparison to noble metals with d-electron-screened plasmons, but retains the important interaction between a collective electron response and an adsorbed molecule. By using two closely spaced hexacene molecules and adjusting the distance between them we match the spectral positions of the electronic resonance of the molecule and the LSPR and thus maximise the plasmon–exciton coupling strength. A satisfactory spectral overlap of the plasmon and molecular resonances (with a slight red shift of the latter with respect to the NP) is achieved for a 2 Å separation between the hexacene molecules. The slightly smaller transition energy of d-hex than the Mg plasmon is deliberate to account for a red shift of the plasmon (similar to refractive index sensing) when the system is assembled. For this fixed d-hex configuration the coupling strength is modified by varying the distance between the NP and d-hex.

We adopt here the approach of Rossi et al. [32, 38] for calculating the generation of hot carriers. The ground state electronic properties of the system are calculated with density functional theory (DFT) using the Perdew–Burke–Ernzerhof (PBE) exchange correlation (XC) functional [42]. The subsequent time-dependent response is calculated with real-time TDDFT (RT-TDDFT) using either the random phase approximation (RPA) or the adiabatic PBE (in the case of time evolution of energy contributions) functionals. The DFT calculations are carried out with the GPAW package [43] using the linear combination of atomic orbitals (LCAO) mode [44] and with uniform real-space grids with the finite difference approximation. TDDFT calculations were conducted using the LCAO-RT-TDDFT implementation in GPAW [45].

The photoabsorption spectra were calculated using the *δ*-kick technique [46] in the linear-response regime and employing the dipole approximation for light–matter interaction. The default projector augmented-wave (PAW) data sets and double-*ζ* polarized (dzp) basis sets [47] provided in GPAW were used for C and H. For Na the corresponding p-valence basis set was used with only one electron in the 3s orbital considered explicitly, while the lower electrons were treated as a frozen core within PAW. The diffuse 3p functions for Na are needed to properly describe the plasmon resonance in the metal nanoparticle [48]. In general, while the used basis sets might not be adequate for yielding numerical values at the complete-basis-set limit, they are expected to be sufficient for the purposes of the present work. For RT-TDDFT a grid spacing parameter of 0.3 Å was chosen to represent densities and potentials, and the molecules/particles were surrounded by a vacuum region of at least 6 Å. The Hartree potential was evaluated on a larger grid with at least 100 Å vacuum around the system and a coarser grid spacing of 1.2 Å, and subsequently refined to the original grid. We used a total propagation time of 45 fs and a time step of 10 as. The spectra were broadened using Gaussian smearing with *σ* = 0.07 eV corresponding to a full width at half-maximum of 0.16 eV.

Subsequently, the system is excited with a Gaussian pulse of the full width at half maximum in frequency space equal to 0.1 eV (see Figure 3(d)) in order to excite only one polariton resonance at a time. The strength of the pulse is small enough, *E*_0_ = 51 μV Å^−1^, to stay in the linear response regime and the field is polarized along the long axis of the molecule. The real-time response to a pulse was calculated as a postprocessing step through a convolution of the frequency response of the system and the pulse as described in Ref. [32]. Before any response calculations, all the isolated systems’ geometries (nanoparticles and single hexacene) were relaxed using the BFGS optimizer in the open-source ASE package [49]. The relaxation calculations used the PBE functional and grid spacing *h* = 0.2 Å. The hexacene dimer and the coupled systems of d-hex and either of the two nanoparticles the were assembled from the individual moieties by setting a particular gap, but not relaxed further.

### 2.2 Metal nanoparticle–molecule coupling

The photoabsorption spectra of the Na_147_ and Na_309_ plasmons and d-hex molecule are plotted in Figure 1(a), illustrating the spectral overlap of the studied systems. Strong coupling between Na_147_ and d-hex is visible in Figure 1(c) as two well-separated lower polariton (LP) and upper polariton (UP), whose positions evolve with the gap size from a value of 3–15 Å. For the largest three gaps ≥25 Å the spectra converge to the incoherent sum of the spectra of Na_147_ and the molecule, indicting the two elements are no longer coupled.

**Figure 2:**
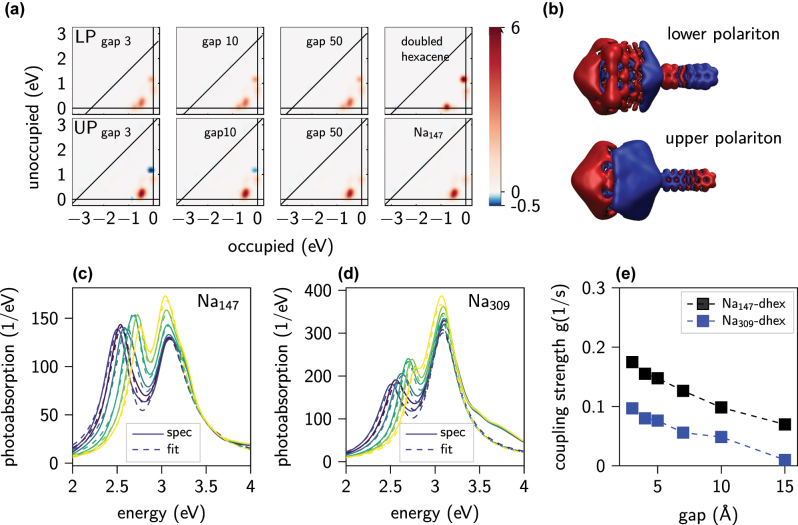
Analysis of coupling between metallic nanoparticle and the molecule. (a) Transition contribution maps to the absorption rates for lower (top) and upper (bottom) polariton resonances. (b) Induced charge density at polariton resonances for a gap of 3 Å. (c) and (d) Absorption spectra and fits of two coupled harmonic oscillators model for Na_147_ and Na_309_, respectively, coupled to the molecule. (e) Effective coupling strength in the system fitted to the classical model as a function of gap width.

**Figure 3:**
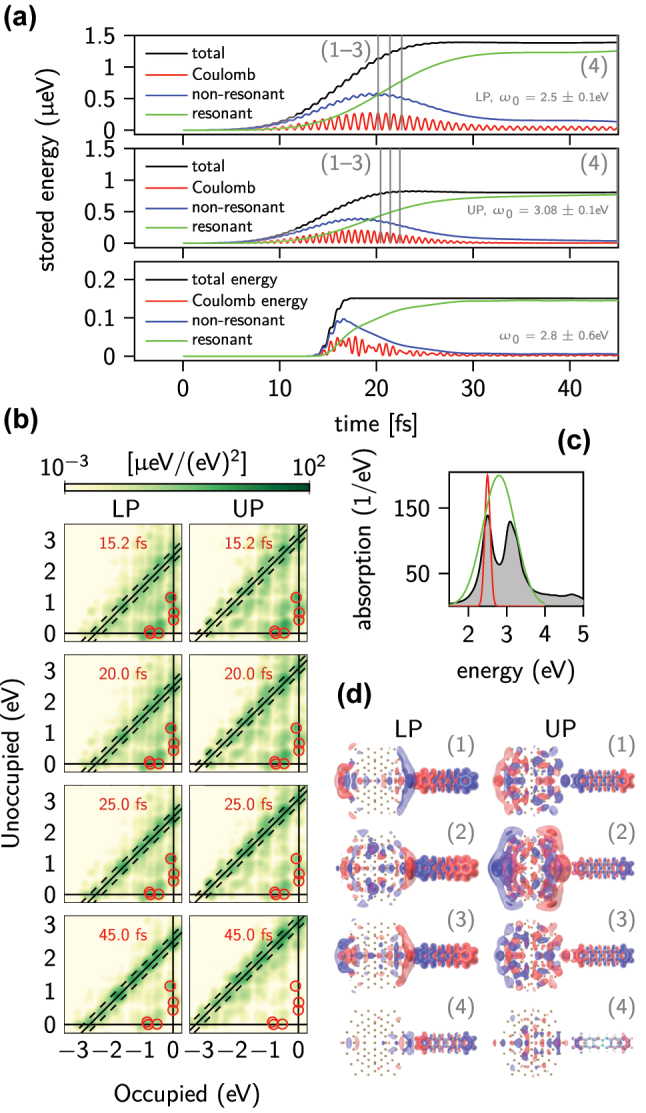
Plasmon decay dynamics. (a) Temporal evolution of stored energy in different forms in a coupled system with a 3 Å gap. The system is excited at the LP (top), UP (middle), and with a broad pulse encompassing both polaritons. (b) Transition contribution maps to energy for selected times for the lower and upper polaritons. The solid line indicates the resonant frequency and the dashed lines indicate frequencies *ω* = *ω*_0_ ± 2*σ*. Red circles denote the molecular transitions. (c) Position and spectral width *σ* of narrow (red) and wide (green) excitation pulses in comparison to an absorption spectrum of the system (shaded area). (d) Induced electron densities in the system at selected moments of time (shown with grey lines in panel (a)) for LP and UP. Two positive (red) and two negative (blue) isosurfaces with values ±2 and ±6 are shown. The values are the same in all panels.

This qualitative change of the spectra from a coupled one with an LP/UP to an uncoupled one for large gaps is seen in Figure 2(a). There we plot the transition contribution maps (TCMs) [50] of absorption at energies corresponding to the lower (top row) and upper (bottom row) polaritons (maxima of the spectra). This allows for a comparison of the systems for a growing gap (and decreasing interaction efficiency) to an isolated Na_147_ cluster and d-hex. The plasmon consists of numerous low energy Kohn–Sham (KS) electron–hole transitions around ∼0.5 eV, while the molecular transitions are visible as intense isolated spots (see the last panel in upper row). The sign of the molecular contributions is opposite for the lower and higher energies, confirming creation of two coupled polariton states. We emphasise here, that while in principle the UP exhibits characteristics of a dark mode, it is nonetheless visible due to a broken symmetry of the plasmon-molecule system. Indeed, at the UP the total stored energy after illumination with a Gaussian pulse is nearly twice lower than in the case of the lower polariton (see Figure 3(a)).

To further confirm that the system is in a strong coupling regime and quantify the macroscopic effective coupling strength, we fit the absorption spectra with a classical model of two coupled harmonic oscillators [51] (see Figure 2(c)–(e)). The fitted effective coupling strength reveals a monotonically increasing value with a decrease of the gap width. As expected, due to a larger volume of the electromagnetic mode, the coupling in the Na_309_-d-hex system is smaller [52]. We included here only systems with gaps smaller than 15 Å, as the splitting of the absorption spectra is not visible for larger ones and the two peaks are, in fact, equal to an incoherent sum of absorption of the two uncoupled spectra. Finally, Figure 2(b) illustrates an exemplary formation of LP/UP for the smallest gap by plotting the induced charge density at the relevant energies, which show, respectively, the bonding and anti-bonding character of the lower and upper polaritons.

### 2.3 Plasmon decay and stored energy contributions

In order to study the hot carrier energy distributions, we first analyse the temporal evolution of energy stored in the system after excitation. This way we can estimate the LSPR dephasing time after which a majority of energy is stored as hot carriers. The excitation pulse (see Figure 3(c) red line), which is in resonance with either of the two polarition branches, induces oscillations of the selected eigenmode of the coupled nanoparticle/molecule, causing the system to go into an excited state. With time, the coherent plasmon/molecule oscillations dephase into single particle excitations. The energy stored in the system can be divided into the Coulomb energy, obtained from the Hartree XC kernel, and electron–hole transition energy contributions [32]. The latter are further analysed with respect to the excitation frequency, specifically transitions resonant with the excitation pulse, i.e. with frequency *ω* = *ω*_0_ ± 2*σ*, (called further as resonant transitions) are considered as being hot carriers. Here, non-resonant transitions are attributed to transitions in d-hex. In the case of noble metals like Ag or Au, non-resonant transitions include also transitions from d-states in the NP which screen the LSPR [32].

In Figure 3(a) we plot the time evolution of stored energy contributions for the lower and upper polaritons in the Na_147_-d-hex system with a 3 Å gap. Although the total energy in the system remains constant (after the impulse ends), the share of different types of electronic excitations varies significantly. Initially, Coulomb energy appears indicating creation of a plasmon, as interaction via the Coulomb force is a characteristic feature of plasmon oscillations [53]. The rapid oscillations reflect energy transformation between the Coulomb energy (maximal for the maximal charge separation) and electric current flow. As time progresses, the plasmon dephases and most of the energy is transferred into resonant transitions [32]. Simultaneously, contributions from molecular transitions also exhibit this dissipation into resonant ones. However, in the case of the LP, the plasmon decay is slower and some Coulomb and non-resonant energy contributions remain visible after 45 fs. Hence, it is possible that even in a strongly-coupled system some of the excitation energy may remain in a molecule-localised quasi-stable non-resonant contribution which is only weakly coupled to other transitions. In practice it may remain as such until other decay mechanisms, such as electron–electron and electron–phonon scattering, absent here, begin to dominate. Note, however, that a polycyclic aromatic hydrocarbon molecule such as d-hex may support collective electron oscillations [54, 55]. Therefore, both parts of the system, i.e. NP and d-hex, contribute to the Coulomb energy and the term plasmon regards here the total system. In the case of the wide spectral excitation pulse (see Figure 3(c) green line), which simultaneously excites both polaritons, we observe additional modulation of the intensity of different energy contributions (see Figure 3(a), bottom panel). These oscillations indicate energy transfer between the coupled NP and molecule, which occurs at time scales shorter than the decay in the system and is a characteristic feature of a strongly coupled system.

Individual transition contributions are plotted in Figure 3(b) as energy TCMs at subsequent moments of time. Initially, non-resonant transitions make up a significant share of all transitions, but after ca. 45 fs a vast majority of them is in resonance with the excitation pulse. The molecular transitions, indicated with red circles, which are clearly visible after excitation, vanish almost completely. Absence of molecular transitions suggests that there is a new decay path for them. We complete the overall picture with plots of induced electron density, see Figure 3(d), at chosen moments of time shown with grey lines in Figure 3(a). The isosurfaces are drawn for the same values of density, respectively for LP and UP. Induced electron density decreases over time (compare panels (1) and (4) in Figure 3(d)), which agrees with the visible reduction of the Coulomb energy maxima and confirms the plasmon decay. At the moments of time corresponding to minima of Coulomb energy (see panels (2) in Figure 3(d)) electron density changes are less pronounced. Interestingly we observe notable surface-to-surface charge separation in d-hex molecule, especially in LP case, indicating the presence of a molecular plasmon.

At longer time scales other decay mechanisms such as electron–electron or electron–phonon scattering, which are not included in the present work, will take place. However, those processes are much slower than the here-studied fast plasmon decay through Landau damping. Electron–electron scattering occurs at time scales of few hundreds of femtoseconds [31, 41] and electron–phonon scattering takes tens of picoseconds [11, 41]. Therefore, the hot carrier distributions presented here should be treated as initial non-thermal distributions before any electron–electron scattering takes place.

### 2.4 Hot carrier energy distribution

**Figure 4:**
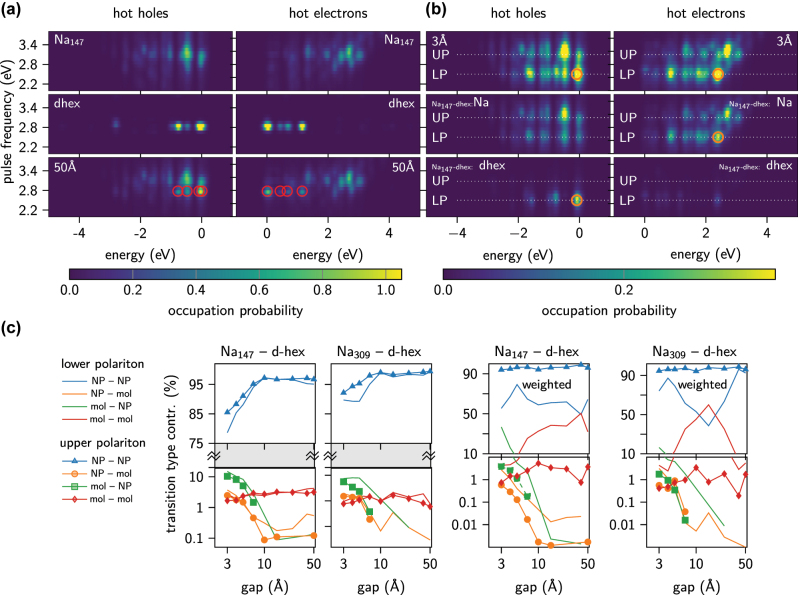
Spectra of and KS transition contributions to hot carriers. (a) Energy distributions of excited hot carriers in isolated Na_147_, d-hex and weakly coupled (gap 50 Å) systems. Red circles indicate the positions of molecular transitions in the molecule. (b) Hot carrier energy distributions in a strongly coupled system with the gap of 3 Å (top) and split into subsystems: Na_147_ and d-hex. Yellow circles indicate the resonant transition from molecular state 187 to NP states described in the text. The zero of energy is set to the Fermi level. (c) Contributions of different transition types. We divide transitions regarding initial and final state localisation (state is of NP/molecular type if weight of Na/d-hex orbitals in pseudo wave function exceeds 50%).

In Figure 4 we present hot carrier energy distributions as a function of the pulse excitation energy assuming a narrow frequency spectrum pulse (0.1 eV). The maxima of hot carrier excitation probabilities follow, respectively, the plasmon resonance and d-hex molecular transition, as plotted in Figure 4(a). As the pulse frequency deviates from the LSPR, Na_147_ exhibits a number of local maxima gradually decaying and the appearance of additional ones with lower intensity. The energy of hot carriers is limited by the energy of the excitation pulse. In contrast, d-hex presents narrow isolated peaks corresponding to discrete energy levels in d-hex. At the bottom of Figure 4(a) we plot the weakly coupled system for the gap of 50 Å. As expected, the charge carrier energy distributions are simply the sums of the analogous distributions for the isolated Na_147_ and the d-hex molecules, the contribution of which are marked with red circles for clarity.

**Figure 5:**
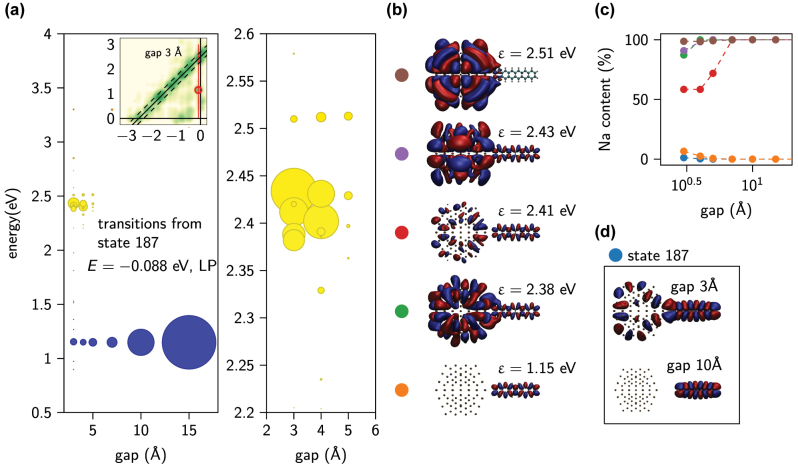
Impact of plasmon–molecule coupling on KS transitions: an example of new nanoparticle–molecule transitions. (a) Transition contributions from the state 187 as a function of gap width between Na NP and molecule. The size of the dot indicates the transition weight. Resonant transitions are marked with yellow dots and non-resonant with blue dots. The inset shows the TCM for Na_147_-d-hex with gap of 3 Å. The red line indicates the energy position of state 187 and the red circle indicates the molecular transition. The excitation pulse frequency is tuned to the position of LP resonance for each gap. (b) Pseudo wave functions for chosen final states of the system with gap of 3 Å. (c) Percentage share of Na atomic orbitals in the pseudo wave function of the final states shown in panel (b) and initial state 187 as function of gap width. (d) Pseudo wave functions of state 187 in the system with gaps of 3 Å (top) and 10 Å (bottom).

In a strongly coupled system for the gap of 3 Å, see Figure 4(b), the energy distribution of hot carriers is no longer a simple sum of contributions from the isolated systems. The main difference is two sets of branches following the LP and UP. Moreover, within the polariton branches the charge carrier energy distributions are markedly different from the ones in the non-interacting system, especially for the LP. This implies activation of different/changed transitions induced by mutual coupling which results in creation of new hybridised NP–molecule states. Using orange circles we denote one of the additional resonant transitions. It generates hot electrons near 2.4 eV and hot holes near −0.088 eV, which is subsequently studied in more detail below and in Figure 5 (circled in the inset in Figure 5(a)). In contrast to the LP, the upper polariton branch exhibits smaller changes of the charge carrier distribution. This is evidenced by a lower percentage of mixed transitions, i.e. from NP to d-hex and d-hex to NP, as plotted in Figure 4(c).

In the bottom two rows of Figure 4(b) we plot the hot electron and hole contributions spatially divided into the Na NP and d-hex with the additional transition, which was mentioned above, being circled. The holes associated with this transition are confined to the d-hex molecules and the electrons to the Na NP. Hence, it is a direct electron transfer pathway between d-hex and Na NP. The energy of these excited hot holes overlaps with one of the active transitions in isolated d-hex and is identified as the molecular occupied state 187.

To clarify the results, we divide all transitions regarding the localisation of initial and final states into molecular, NP and mixed ones (including transitions form NP to d-hex and-hex to NP. We define here, that the transition is of NP/d-hex type, if the share of Na/d-hex orbitals exceeds 50%. The share of each type is plotted in Figure 4(c) as a function of gap width between NP and d-hex for both polariton branches. The share of mixed transitions visibly grows with a decreasing gap size (and thus increasing coupling strength) below ca. 10 Å. This increase is smaller for the Na_309_-d-hex system, which is characterised by a lower coupling strength for the same gap size (Figure 2(e)) due to an increased mode volume of the cavity, which for small single nanoparticles can be approximated by the geometrical volume [52]. The dominant mixed transitions are those originating in a molecular state and finishing in an NP state (green line in Figure 4(c)). When accounting for the transition weight, cf. the two panels on the right of Figure 4(c), it is clear that the mixed transitions are more active in the LP than in the UP; despite they are approximately equal in numbers (cf. left panels of Figure 4(c)). Hence, the significant intensity of the mixed transitions markedly alters the energy distribution of hot carriers at the LP.

In Figure 5 we discuss the evolution of transition weights (from the chosen state 187 whose electronic density localised at the molecule is one of the initial states of an active transition of isolated d-hex) with a changing coupling strength between the subsystems due to different gap sizes. Each transition is marked with a circle whose diameter is proportional to its relative strength and colored yellow to denote resonant transitions or blue for non-resonant ones. The excitation pulse frequency is tuned to the position of LP resonance for each gap and the pulse width is equal to 0.1 eV. For wide gaps, ≥7 Å, dominant is an intra-molecular transition to an unoccupied molecular state (marked orange in Figure 5(b)) of energy 1.15 eV. With a decreasing gap its share decreases and for gaps ≤5 Å new resonant transitions appear. The plots of pseudo wave functions of the new final states (see Figure 5(b)) reveal all of them to be partially hybridised between the molecule and NP. In particular, there is a non-zero probability of finding an electron in the gap between NP and d-hex. In Figure 5(c) we plot the percentage share of Na atomic orbitals in the pseudo wave functions for the presented states as a function of gap size. For a decreasing gap the share of Na orbitals increases in states which were initially molecular ones and, correspondingly, decreases in those which were initially NP-like. This is exemplified in Figure 5(d) for the occupied state 187, whose pseudo wave function for a gap of 10 Å is fully localized over the molecule, but for a gap of 3 Å is hybridized over the whole NP-molecule system.

**Figure 6:**
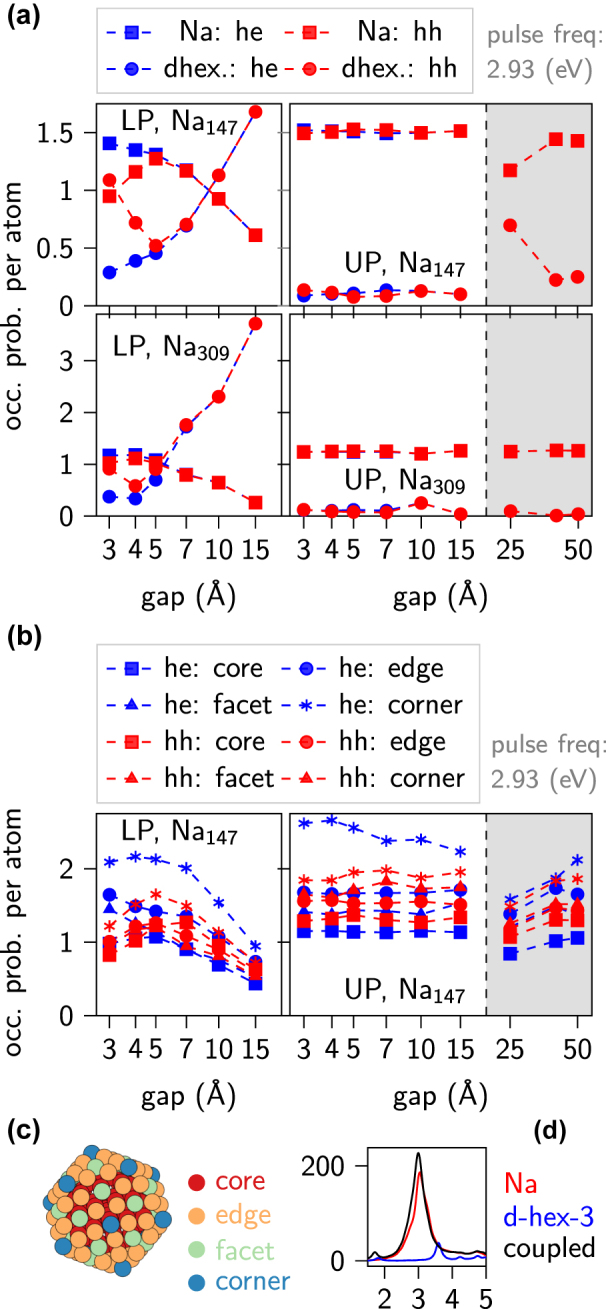
Hot carrier generation efficiency and electron transfer between nanoparticle and molecule. (a) Probabilities of hot carrier excitation per atom separated into Na and d-hex contributions as a function of the gap. Top row: Na_147_, lower row: Na_309_. The grey background indicates gaps for which the system is quasi-uncoupled. The excitation pulse energy corresponds to the frequency of the LP or UP. Normalisation: a value of 1 corresponds to a uniform distribution of carriers within the system. (b) Probabilities of hot carrier generation in various parts of Na_147_. (c) Scheme of Na_147_ showing division into different types of atomic sites. (d) Absorption spectrum for Na_147_ coupled to d-hex with a separation between hexacene molecules of 3 Å (electronic transition at 3.6 eV does not overlap with the LSPRs of both Na_147_ and Na_309_. Splitting of the plotted absorption spectrum is not observed even for a gap between the NP and molecule of 3 Å, nor is charge carrier transfer between the subsystems.

Further insight may be gained by evaluating the per-atom hot carrier occupation probability at a particular atomic site. Those probabilities are presented in Figure 6 as a function of the NP–molecule gap for both the Na_147_ and Na_309_ clusters. The probability is normalised to a uniform distribution of hot carriers over the whole system. The frequency of the excitation pulse is matched to the peaks of the LP and UP for gaps ≤15 Å. For larger gaps the NP and molecule are effectively uncoupled and the absorption peaks correspond to their individual resonances. Therefore, for those gaps the system is excited with a pulse frequency *ω*_0_ = 2.93 ± 0.1 eV centred at the mean energy of the LP and UP for the 15 Å gap.

The carrier occupation probability versus gap size, equivalently coupling strength, is markedly different for both polaritons. At the LP two regimes are identified. For systems with gaps larger than 7 Å, whose coupling strength is comparatively small, the occupation probability of hot electrons and hot holes in each subsystem (NP or d-hex) individually is the same. However, with an increasing gap the probability to observe the excitation in the molecule increases at the expense of the NP. This suggests that the excitation strength of each subsystem changes, possibly due to changes of the local electromagnetic field enhancement. Indeed, for the intermediate gaps of 7–15 Å the field enhancement around the molecule is quite significant. Conversely, for gaps below 7 Å the hot electron and hole occupation probabilities in each subsystem individually diverge. For the molecule a significant, ca. 3-fold excess of hot holes is found. Simultaneously in the NP more hot electrons are present. This indicates that there is a hot electron transfer from d-hex to the NP and this charge transfer is inversely proportional to the size of the gap. At these smaller gaps, despite the field enhancement provided by the plasmon being larger [52], the overall probability of generating charge carriers in the molecule is smaller. This is because for small gaps the collective plasmon mode is instead inducing hybridization of the NP and molecular states, which leads to the aforementioned hot electron/hole distributions and charge transfer. These observations for the coupled system with Na_147_ are also present for the larger metal cluster, although the probability of hot carrier generation in the molecule for intermediate gaps (7–15 Å) is larger when coupled to the Na_309_ NP. The cause of this additional enhancement is the larger size of the NP and the comparatively longer decay length of the plasmon, which in turn translates into a larger average field enhancement over the molecule.

**Figure 7:**
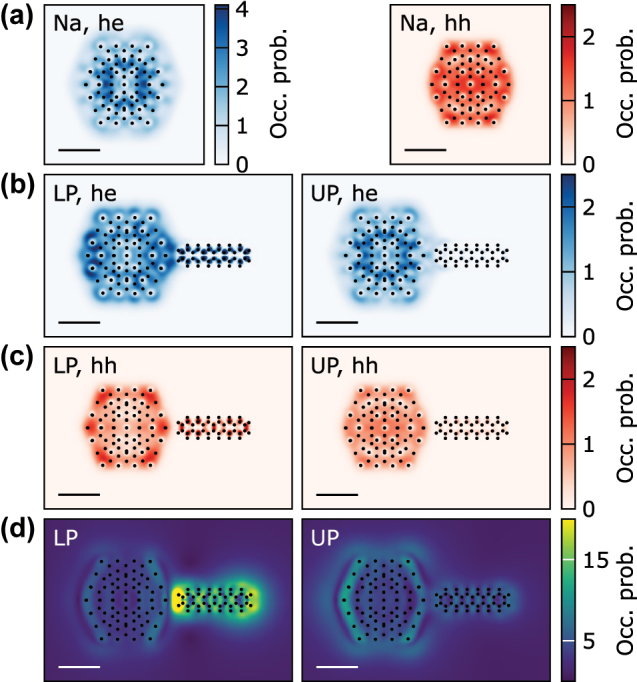
Comparison of spatial distribution of hot carriers and induced electric field. (a) Spatial distribution of hot electrons (he) and holes (hh) in Na_147_ at the plasmon resonance. (b) and (c) Spatial distribution of hot electrons and holes in Na_147_-d-hex with of 3 Å for LP (left) and UP (right) polariton frequencies. Note the significant changes of the hot carrier distributions in the NP when strongly coupled to the molecule. (d) Enhancement of electromagnetic field shows a very strongly enhanced field around the molecule at the LP and a comparatively weaker at the UP. The scale bars in all panels are the same and equal 10 Å.

The charge carrier generation under illumination of the upper polariton is significantly different from the LP case. A majority of the hot carriers is generated inside the NP and equal probabilities of finding hot electrons and holes inside the individual subsystems indicate that there is no significant charge transfer between them, also for the smallest gaps. This difference in the rates is probably a result of unequal excitation of the NP and d-hex. This hypothesis is supported by electromagnetic field enhancement distributions plotted in Figure 7(d). Indeed, the amplitude of the field enhancement in the molecule at the UP is smaller than in the NP.

In Figure 6(b) we show the hot carrier occupation probabilities per atom at various atom positions inside Na_147_ while coupled to the molecule. Both electron and hole occupation probabilities for corner-sites and edge-sites are visibly higher than in the case of an uniform distribution. This is seen for both the LP and UP. Moreover, in both cases, the probability of finding an electron at these sites is higher than the probability of finding a hole. The opposite case is for core and facet-sites. Here it is more probable to find a hot hole than a hot electron. Therefore, part of the additional electrons in corner and edge-sites comes from a transfer from core and facet-sites [32], however, in part this inequality is caused by the presence of the molecule, as the probability of finding hot electrons at the corner sites is larger than for an uncoupled NP with a large gap ( ≥25 Å).

In order to test weather this effect is a result of strong coupling, we calculated hot carrier generation probabilities in a system of Na_147_ coupled to a dimer of hexacene molecules, but with the distance between hexacenes equal to 3 Å (instead of 2 Å). The electronic transition of this modified hexacene dimer is thus spectrally shifted to the blue with respect to the LSPR of the NP and does not exhibit strong coupling (Figure 6(d)). We have found that the carrier transfer between Na NP and d-hex molecules is negligible as the difference in occupation probability for hot electrons and hot holes both in Na NP and d-hex molecules is smaller than 0.01.

In Figure 7 we compare the spatial distribution of hot carriers (of energy higher than 1 eV for hot electrons and −1 eV for hot holes) with the electromagnetic field enhancement. Hot electrons, in contrast to hot holes, are spread relatively far outside the NP. The symmetric distribution observed for an isolated NP, is significantly modified due to the presence of d-hex molecule. In the case of the LP excitation, differences are significant both for electrons and holes, while in the case of UP excitation the hot hole distribution in a coupled NP is similar to the one for an isolated NP. Generally, in a coupled system, hot carriers are localised preferably near the NP surface and homogeneously inside the d-hex. Interestingly, there is a significant occupation probability for hot electrons in the gap between NP and d-hex. This result agrees with calculated contributions from pseudo wave functions of the new hybridised states (shown in Figure 5(b)). Localisation of hot carriers in these hybridised states is consistent with a number of works on hybrid plasmonics materials, which reported predominant generation of hot carriers at the interface between a plasmonic and a non-plasmonic material [18]. Although there is no straightforward relation between the field enhancement and hot carrier distributions, in the gap between the NP and the molecule one notices similarities between the spatial distributions of the electric field and the hot electrons. Furthermore, a much higher field enhancement in d-hex at the LP correlates with higher hot carriers occupation probabilities. This supports the existence of a correlation between the field enhancement and the hot carrier generation rate, though it is only one of the variables influencing the generation of hot carriers.

## 3 Conclusions

By studying interaction between the plasmon resonance of a sodium nanoparticle and an electronic transition in an exemplary molecular system we have shown there is a possibility to manipulate the energy and the spatial distribution of generated hot carriers by tuning the degree of strong coupling between the subsystems. The energy distribution of hot carriers differs significantly from the case of an uncoupled system; in particular the energy of generated hot carriers may be higher when the system is coupled. This change arises from an appearance of new hybridised NP–molecular states, and is facilitated by the presence of a collective plasmon spectrally tuned to the molecular exciton. This allows for a new set of transitions in the system, especially transitions between primarily molecular and NP states. As a result, the number of possible decay paths for molecular and NP transitions increases [15]. Interestingly, for small gaps (here ≤5 Å) it is possible to observe charge transfer between two coupled subsystems, in contrast to a case in which the molecular exciton is spectrally detuned from the plasmon.

The modification of the spatial distribution of hot carriers is most pronounced in the vicinity of the NP surface, where the density of hot carriers is significantly increased. In the case of the system with the gap equal to 3 Å, hot electrons can be also found in the gap between the NP and d-hex. This may ease extraction of hot carriers, especially since they are localised close to the donor surface.

Moreover, strong coupling can be used to adjust the optimal excitation frequency for hot carrier generation, to either match the energies of the carriers for use in promising reactions or to match the spectrum of an abundant light source. Intense energy transfer between the subsystems causes creation of hybridised plasmon–exciton states with new resonant frequencies. We have shown that hot carriers generation rates reach their maxima at those new resonant frequencies. Finally, the hot carrier generation rate does not change significantly with the coupling strength, but rather scales with the intensity of the external exciting electromagnetic field and internal field enhancements generated by the interacting subsystems.
